# Distribution of basal membrane complex components in elongating lens fibers

**Published:** 2008-06-30

**Authors:** Jeffrey Y. Lu, Tabraiz A. Mohammed, Sean T. Donohue, Kristin J. Al-Ghoul

**Affiliations:** 1 Departments of Anatomy & Cell Biology, Rush University Medical Center, Chicago, IL;; 2 Department of Ophthalmology, Rush University Medical Center, Chicago, IL

## Abstract

Purpose: To localize specific components of the Basal Membrane Complex (BMC) of elongating lens fibers at defined points in their migration to the posterior sutures.

Methods: Normal, juvenile (4-6 week old) Sprague-Dawley rat lenses (n=46) were utilized. Lenses were either decapsulated to obtain whole mounts of lens capsules or sectioned with a vibrating knife microtome. Sections (100 µm thick) were cut parallel to the equatorial plane, beginning at the posterior pole. On both sections and whole mounts, F-actin was localized using phalloidin-FITC while myosin, cadherins, and β1 integrin were localized using immunofluorescent labeling. Specimens were visualized on a laser scanning confocal microscope.

Results: F-actin labeling in the equatorial and peri-sutural regions was predominately localized to the periphery of basal fiber ends (consistent with our prior results). At sutures, labeling for F-actin in the BMC was rearranged into numerous small profiles. Furthermore, labeling intensity for F-actin was increased at sutures. Myosin was present in the BMC in all locations examined as a diffuse plaque at fiber ends. Similarly, β1 integrin was also distributed throughout the BMC within the actin-rich borders in all regions except adjacent to and at the suture branches. In that location immunofluorescence for β1 integrin appeared to be reduced. In the equatorial, lateral-posterior, and peri-sutural regions, cadherin showed strong localization around the periphery of basal fiber ends. However, cadherin labeling was markedly reduced in the BMC as fibers detached from the capsule and abutted to form sutures (i.e. in the sutural region). Cadherin was concentrated along the short faces of elongating fiber mid-segments.

Conclusions: It appears that F-actin, cadherin and β1 integrin components of the BMC undergo controlled rearrangements in the final stages of migration and detachment from the capsule.

## Introduction

Elongation of nascent lens fibers involves the migration of basal fiber ends along the lens capsule from the meridional rows at the equator to their sutural destinations on the lens posterior. The interaction of migrating basal fiber ends with the capsule is mediated by the basal membrane complex (BMC). The BMC is defined as the fiber cell basal membrane, its resident integral membrane proteins, and their associated cytoskeletal and regulatory elements. In avian lenses, BMC components include integrins, cadherins, actin, myosin, caldesmon, paxillin, focal adhesion kinase (FAK), and myosin light chain kinase (MLCK) [1].

Components of the BMC, such as actin, β1 integrin, and N-cadherin, have been studied to elucidate their role in cell attachment to the extra cellular matrix (ECM), proliferation, and migration. It is well established that β1 integrins are the primary integrin receptor for the basal lamina proteins of the lens capsule [2]. These integrins function as bidirectional signaling receptors, mediating cytoskeletal-ECM interactions that impact fiber adhesion and polarization [3]. The cell adhesion molecule, N-cadherin, also plays a role in lens fiber differentiation, specifically regulating differentiation-dependent cytoskeletal reorganization via its linkage to actin [4]. Cadherins are also essential to the systematic formation and organization of cell-cell interactions during migration [5]. It is therefore not surprising that N-cadherin has been co-localized with actin both in the native BMC and in cell culture [1,4]. Actin has been found to play a key role in coordinating structural changes and maintaining the integrity of lens development during fiber cell elongation [6-10].

During fiber migration, fiber ends must reach precise destinations, detach from the capsule at the appropriate time, and overlap and/or abut with opposing fiber ends. This convergence of opposing fiber ends results in the formation of lens sutures. Although fibers differentiate and elongate to form sutures in a similar manner in all vertebrate lenses, their migration patterns are not identical, leading to variations in sutural anatomy among species. Specifically, there are four distinct suture patterns: branchless or umbilical sutures, line sutures, Y sutures, and star sutures (see [11] for a detailed review). In avian and reptile lenses, fiber ends migrate essentially straight toward the poles where they detach from the capsule (posteriorly) or epithelium (anteriorly) to overlap and abut. This growth scheme results in a system in which all fibers are meridians, and branchless (umbilical) sutures are formed.

In all other vertebrate lenses, elongating fiber ends within each growth shell have diverse migration paths, resulting in fibers of variable length and curvature, which overlap and abut to form branched sutures. These lenses have two basic types of fibers, those with straight end segments (straight fibers) and those with curved end segments (curved fibers). Straight fibers extend to a pole on one end and to the proximal end of a suture branch on the other end. Curved fibers lie between the straight fibers. The simplest branched suture pattern is formed when fiber ends (of curved fibers) migrate in one of four directions, resulting in a line suture wherein the two branches are oriented 180° apart. These are seen in rabbit and amphibian lenses. Similarly, in lenses with Y sutures, fiber ends within a growth shell migrate in one of six directions to form a three-branched, “Y” pattern. Y sutures are found in rodent, feline, canine, porcine, ovine, and bovine lenses. Finally, when fiber ends in each growth shell migrate in one of twelve directions, a six-branched, simple star suture pattern results. This simple star pattern is characteristic of primate lenses and, in humans, becomes increasingly complex with age. Each of the four suture patterns has a specific effect on lens optical quality as measured by focal ability [12].

From the above, it can be seen that proper formation of lens sutures is crucial in establishing and maintaining structural order (at the cellular level), which minimizes large particle scatter thus promoting transparency [13]. Furthermore, because defined suture patterns are only formed via coordinated fiber end migration, it is clear that migration is a key process in producing appropriate fiber organization. In addition to maximizing lens clarity, structural order is known to impact lens function. Specifically, a number of studies have demonstrated that excessive disorganization of newly elongated fibers and lens suture patterns results in a degradation of lens focal ability [14-16] and if extreme, may compromise lens transparency [17,18].

While it is clear that both the anterior and posterior sutures exert a negative effect on the transparency and refractive properties of lenses, the process of posterior suture formation appears particularly vulnerable to compromise leading to excessive disorder and eventual cataract [19,20]. In fact, prior structural analyses have suggested that faulty fiber migration wherein the posterior sutures fail to form results in posterior subcapsular cataract (PSC) formation in several animal models [21-26]. Thus, it is essential to gain an understanding of all aspects of basal fiber end migration including morphology, organization, migration patterns and the identity and distribution of BMC components, in order to evaluate how and why this process is disrupted in pathological situations.

Although the distribution of several BMC molecules was described in fibers at the beginning of elongation [1], little is known about their molecular arrangement during the terminal phases of migration and formation of lens sutures. Our recent investigations have shown that the morphology and organization of basal fiber ends in mammalian lenses displays significant variation during migration, especially during the terminal phases [27]. Additionally, BMC architecture in mammalian lenses, which feature branched suture patterns, may be substantially different from that reported for avian lenses, which have branchless, umbilical sutures. Therefore, the goal of this study was to localize specific BMC components in rodent elongating lens fibers at both the initial and terminal stages of fiber end migration. Our results indicate that the distribution of several BMC components alters as fiber ends approach their sutural destinations.

## Methods

### Lenses

Normal Sprague-Dawley rats (4-8 weeks old) were utilized for this investigation. All animals were handled in compliance with the ARVO Statement for the Use of Animals in Ophthalmic and Vision Research and with Rush University Medical Center IACUC (Institutional Animal Care and Use Committee) guidelines. A total of 48 animals were sacrificed by intraperitoneal injection of a euthanasia agent, and the eyes were immediately enucleated.

### Whole-mount lens capsules

Whole mounts of the posterior lens capsule were prepared as previously described [27]. Briefly, lenses were pre-fixed for 2 min in 2% paraformaldehyde, washed in 0.07 M phosphate buffer saline (PBS), and placed on dental wax with the posterior surface down. The capsule was lightly grasped near the anterior pole, then carefully peeled away from the lens and pinned to the dental wax. The decapsulated fiber mass was gently removed and the capsule (with the sheared-off fiber ends adhered) was fixed for 30 min in 3% paraformaldehyde in 0.07 M (PBS). This procedure generally yields an intact posterior lens capsule with attached basal fiber ends in the initial regions of fiber end migration (defined previously [27]), specifically the equatorial region and the proximal portion of the posterior lateral region. Basal ends in more distal locations do not readily remain adhered to the rat lens capsule, even after the pre-fixation procedure.

### Vibratome sectioning

In order to examine the distribution of BMC components in fiber ends distal to the equator, posterior polar sections were utilized. Sections have the additional advantage of yielding intact fiber ends, whereas the capsule stripping method may introduce mild artifacts in fiber end morphology due to tension created during the stripping process (wherein fiber ends are sheared off from the remainder of the cells).

**Figure 1:**
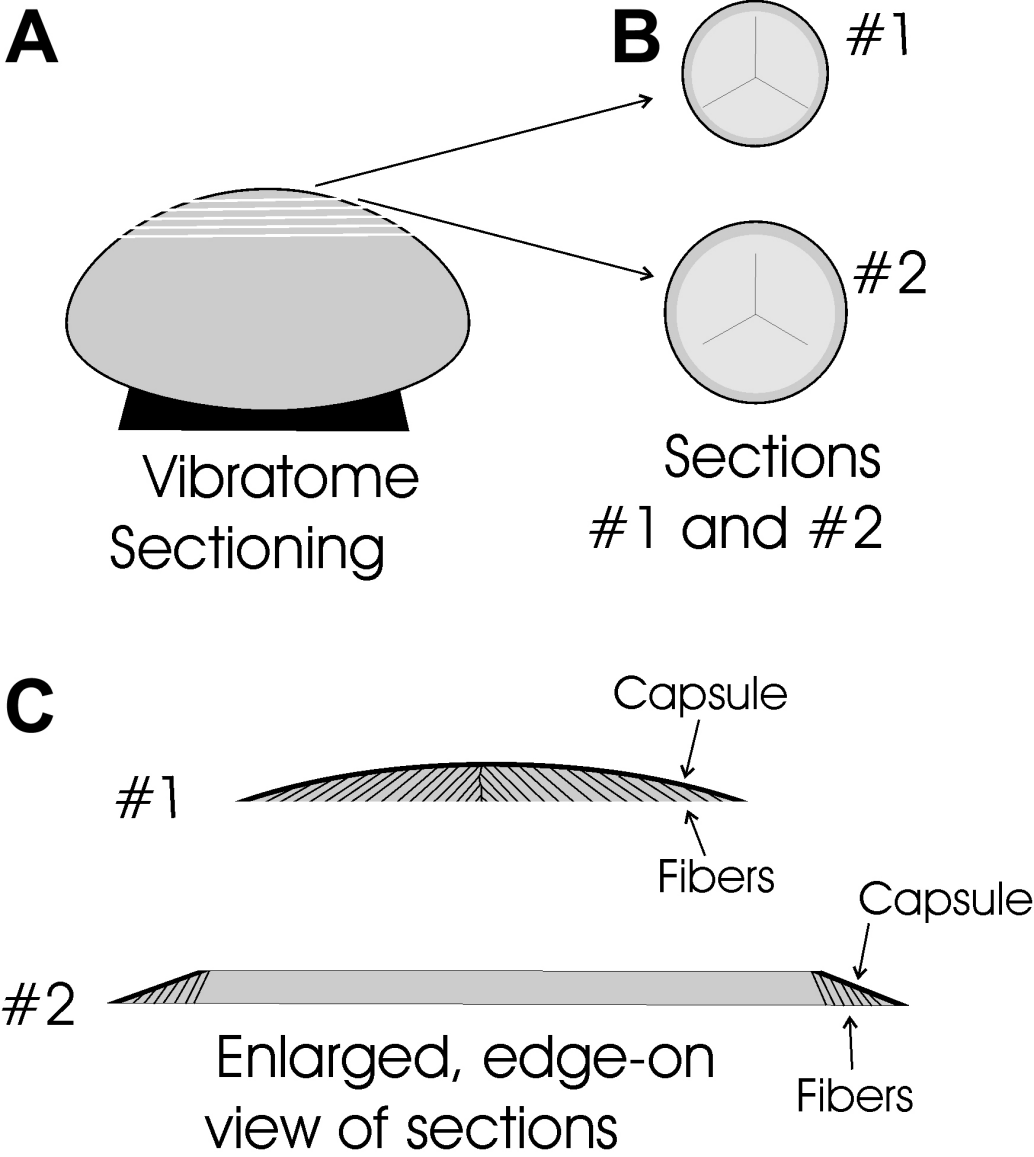
Diagrammatic representation of the vibratome sectioning technique. **A**: Lenses were mounted on the anterior surface and sectioned beginning at the posterior pole. **B**: When viewed face-on, all sections contain portions of the suture planes. Typically the first 1-3 sections contain elongating fiber ends in the peri-sutural and sutural regions. **C**: An enlarged, edge-on view of the first two sections demonstrates that migrating fiber ends are present beneath the entire curved surface of the capsule in section #1. However, in section #2 (and all subsequent sections) elongating fiber ends are present only beneath the beveled edges of sections covered by capsule.

Before sectioning, the posterior retina and sclera were removed and the remainder of the eye was placed in 3% paraformaldehyde in 0.07 M PBS. After fixation for 2 h, the lenses were dissected from the eye and briefly rinsed in 0.07 M PBS. In order to obtain posterior sections, lenses were mounted on a sectioning chuck, anterior side down, with cyanoacrylate glue (Figure 1A) and embedded in warm 2% agar, which solidified at room temperature. Lenses were sectioned parallel to the equator with a vibrating knife microtome (Lancer Series 1000 or Vibratome 3000 Series; Vibratome, St. Louis, MO). Sections 100 μm thick were cut beginning at the posterior pole (Figure 1B) using an amplitude of 3 and a speed of 5 (approximately 0.6 mm/sec). Sections were fixed for an additional 30 min in 3% paraformaldehyde, briefly washed in 0.07 M PBS, and then processed for immunocytochemistry.

Ideally, the first vibratome section should yield the enveloping posterior capsule, the posterior fiber ends of elongating fibers, and the nascent suture branches, as well as the underlying growth shells of cortical maturing fibers. Fiber “feet” should be seen in all locations beneath the rounded, uncut posterior lens surface (Figure 1C; #1). In subsequent sections, fiber feet should be present under the capsule only beneath the beveled edge of the section (Figure 1C; #2). In some instances sections were retrieved at or near the equator of the lens by continuing to section deeper into the specimen.

### Antibodies

Rabbit antibody to myosin IIA (non-muscle, M 8064; Sigma Chemical Company, St. Louis, MO) was utilized at a dilution of 1:100. Goat polyclonal antibody to β-1 integrin (N-20; Santa Cruz Biotechnology, Inc., Santa Cruz, CA.) was used at a dilution of 1:200. Goat polyclonal antibody to N-cadherin (N-19; Santa Cruz Biotechnology, Inc.) and mouse monoclonal anti-pan cadherin (clone CH-19; Sigma Chemical Company) were both utilized at a dilution of 1:200. Goat polyclonal antibody to actin (I-19; Santa Cruz Biotechnology, Inc.) was utilized at a dilution of 1:200. Mouse anti-fodrin monoclonal antibody was used at a dilution of 1:200 (MAB1685; Chemicon International, Temecula, CA). Goat anti-rabbit-TRITC antibody (Molecular Probes, Inc., Eugene, OR) was used at a dilution of 1:100. Rabbit anti-goat-FITC antibody, rabbit anti-goat-TRITC antibody, rabbit anti-mouse FITC antibody and rabbit anti-mouse TRITC antibody (Sigma Chemical Company) were all utilized at a dilution of 1:100. Donkey anti-mouse-AMCA antibody, donkey anti-rabbit-FITC, and donkey anti-goat-TRITC (Jackson ImmunoResearch, West Grove, PA) were also utilized at dilutions of 1:100 for double and triple labeling.

### Immunocytochemistry

Immunolabeling was performed by sequentially immersing sections in drops of solution as follows. Sections were permeabilized in 0.2% Triton X-100 for 30 min followed by a 1 h incubation in a blocking solution of 10% goat serum (for N-cadherin, pan-cadherin and myosin IIA labeling), 10% rabbit serum (for β-1 integrin labeling), or 10% Donkey Serum (for actin I-19, myosin IIA, and pan-cadherin multiple labeling), 1% bovine serum albumin (BSA) and 0.05% thimerosal in 0.07 M PBS. The sections were then reacted with a primary antibody specific to the BMC component of interest (actin, β-1 integrin, N-cadherin, pan-cadherin, or myosin IIA) for at least 2 h at room temperature or overnight at 4º C.  Primary antibodies from different host animals were combined into a single solution during this step for multiple labeling experiments (triple labeling). Appropriate primary antibody dilutions were determined by dilution series tests; dilutions were made in the blocking solution. Following three 10 min washes in blocking solution, sections were incubated in a secondary antibody conjugated to a fluorescent label (FITC, TRITC, AMCA, or a combination of each) for at least 2 h at room temperature. Sections were washed 4 times in 0.07 M PBS for 10 min each, then mounted on glass slides (Gold Seal Products, Portsmouth, NH) with Vectashield mounting medium (Vector Laboratories, Inc., Burlington, CA) to prevent photo bleaching. Specimens were examined on either a Zeiss LSM 510 or Zeiss LSM 510 Meta laser scanning confocal microscope (Research Resource Center, University of Illinois at Chicago).

In addition to the immunolabeling of actin described above, F-actin was also localized using fluorescent-labeled phalloidin. Fixed lens sections were permeabilized in 0.2% Triton X-100 for 30 min, then incubated with phalloidin conjugated to either fluorescein isothiocyanate or tetramethyl rhodamine isothiocyanate (Sigma Chemical Company) at a dilution of 1:100 of a methanolic stock (200 U/ml) solution. Sections were washed 4 times in 0.07 M PBS for 10 min each, then mounted and examined as detailed above.

Control experiments were conducted to ascertain whether the fluorescence was specific binding or non-specific background. Lenses were dissected and processed as previously described. However, normal (non-immune) serum from the same species in which the primary antibody was raised was utilized in place of the primary antibody as follows: for myosin labeling, sections were treated with normal rabbit serum; for ß-1 integrin and N-cadherin labeling, antibody was substituted with normal goat serum; for pan-cadherin labeling, normal mouse serum was used. Following incubation, the specimens were processed and viewed as described above.

### Detergent extraction

To assess whether the antigenic sites of some BMC components (specifically cadherin and ß-1 integrin) were masked by changes in molecular interactions during fiber elongation, some sections were subjected to detergent extraction to ‘unmask’ antigens. Variations of this technique have been successfully used in the lens to reveal epitopes that were either inaccessible or masked during protein rearrangements as a consequence of fiber differentiation [28,29]. In the present study the method described by Beebe et al. [29] was utilized with minor modifications. Specifically, fresh, unfixed lenses were mounted with cyanoacrylate glue, embedded in 2% agar and sectioned with a sapphire knife at a thickness of 300 µm. Sections were immediately placed in detergent buffer (0.5% Nonidet P 40 Substitute, 100 mM KCl, 5 mM MgCl_2_, 1 mM EDTA disodium salt, 2 mM 2-mercaptoethanol, and protease inhibitor cocktail) for 3 h at 4°C. Following extraction, sections were fixed and immunolabeled as described above.

### Data analysis

To locate and confirm the position of nascent suture branches, paired confocal and differential interference contrast (DIC) images were collected from most specimens. Our previous definitions of the regions of fiber end migration were utilized for this assessment [27]. To summarize, the 4 regions of basal fiber end migration are: 1) equatorial, 2) lateral-posterior (posterior from the equator to within 150 µm of sutures), 3) peri-sutural (150 µm surrounding the sutures), and 4) sutural. In order to thoroughly characterize BMC components, both single-plane and z-series’ were collected at 25X, 40X, and 60X magnification, at a pinhole of 1.0. Images were viewed and analyzed using the Zeiss LSM Image Browser version 2.30.011 (Carl Zeiss, Jena, Germany) and Adobe Photoshop version 7.0 (Adobe Systems Inc, San Jose, CA).

To evaluate changes in labeling intensity, optical density line scans were made across digital confocal images and plots were generated using Scion Image v. beta 4.0.2 (Scion Corp., Frederick, MD). Line scans were oriented 90º to suture branches and included both the sutural and adjacent peri-sutural regions. Plots were inverted to show high labeling signal as peaks and low labeling signal as troughs.

## Results

**Figure 2:**
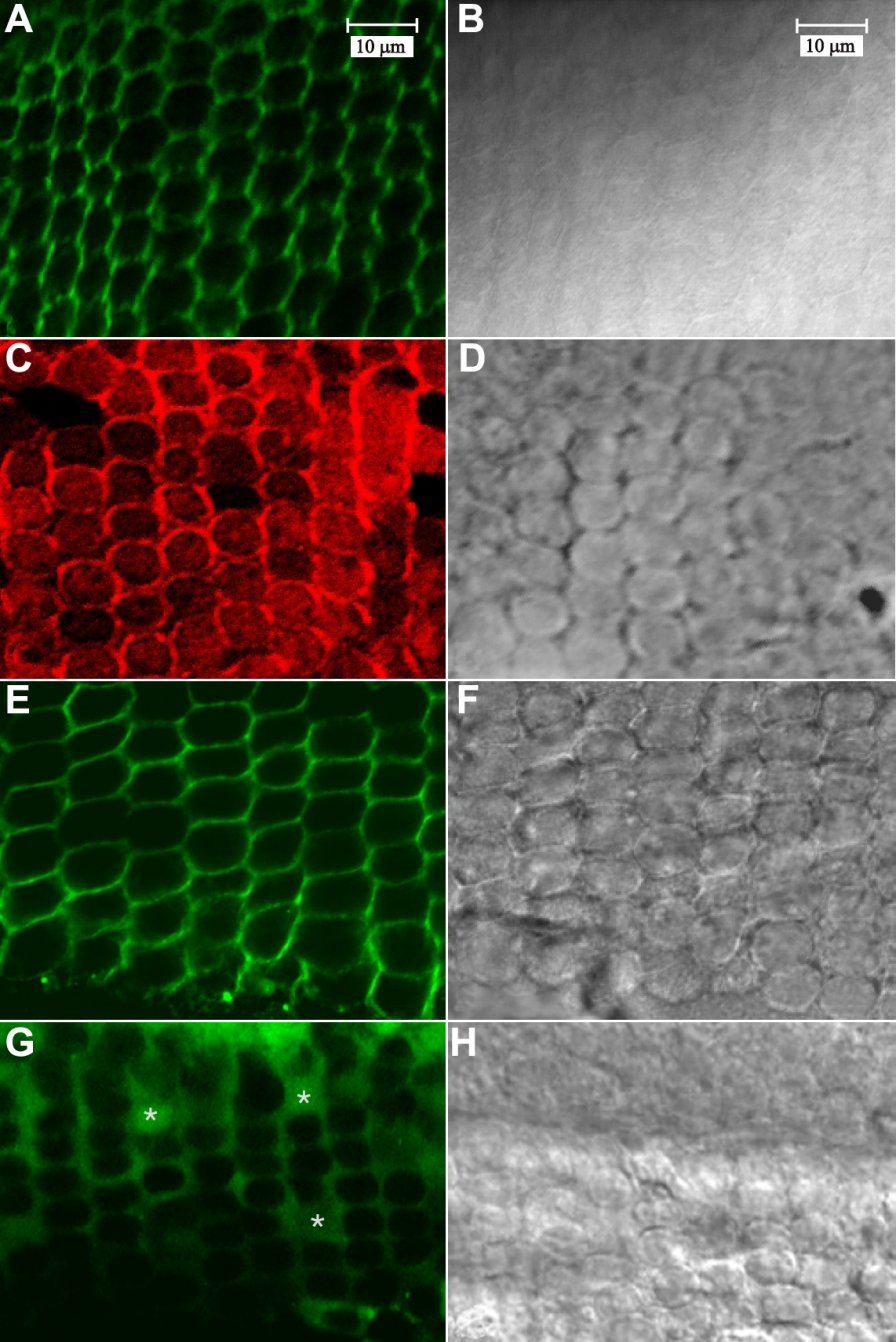
LSCM visualization of BMC components in whole-mount lens capsules at the equatorial region. **A**-**B**: Paired fluorescent (**A**) and DIC (**B**) images of F-actin labeled with phalloidin-FITC. F-actin is concentrated at cell borders. **C**-**D**: Paired fluorescent (**C**) and DIC (**D**) images of (non-muscle) myosin IIA labeling. Myosin is distributed throughout the BMC and enriched at the cell periphery. **E**-**F**: Paired fluorescent (**E**) and DIC (**F**) images of pan-cadherin labeling. As expected, cadherin was localized to lateral membranes. **G**-**H**: Paired fluorescent (**G**) and DIC (**H**) images of β1 integrin labeling. Distribution of β1 integrin label was diffuse, forming a plaque at the basal portion of the cells (asterisks). Micrographs **A**-**H** are at equivalent magnifications.

The distribution of selected BMC components was first established in the equatorial region using whole-mount lens capsules (Figure 2). As previously reported, [27] F-actin (visualized with phalloidin-FITC) predominantly localized to the periphery of the BMC with only faint actin fluorescence present within the brighter profiles (Figure 2A,B). Labeling for myosin IIA also showed strong peripheral fluorescence as well as definitive labeling within the remainder of the BMC (Figure 2C,D). Cadherin distribution in the equatorial region was assessed with a pan-cadherin antibody and demonstrated the expected localization at lateral (cell-cell) borders of the BMC (Figure 2E,F). Not surprisingly, cadherin immunofluorescence was not detected at the basal membrane (where cells interface with the capsule). Labeling for β-1 integrin in equatorial fibers was diffuse and present as a basal plaque (Figure 2G; asterisks) as well as prominent at lateral cell borders (Figure 2G,H).

**Figure 3:**
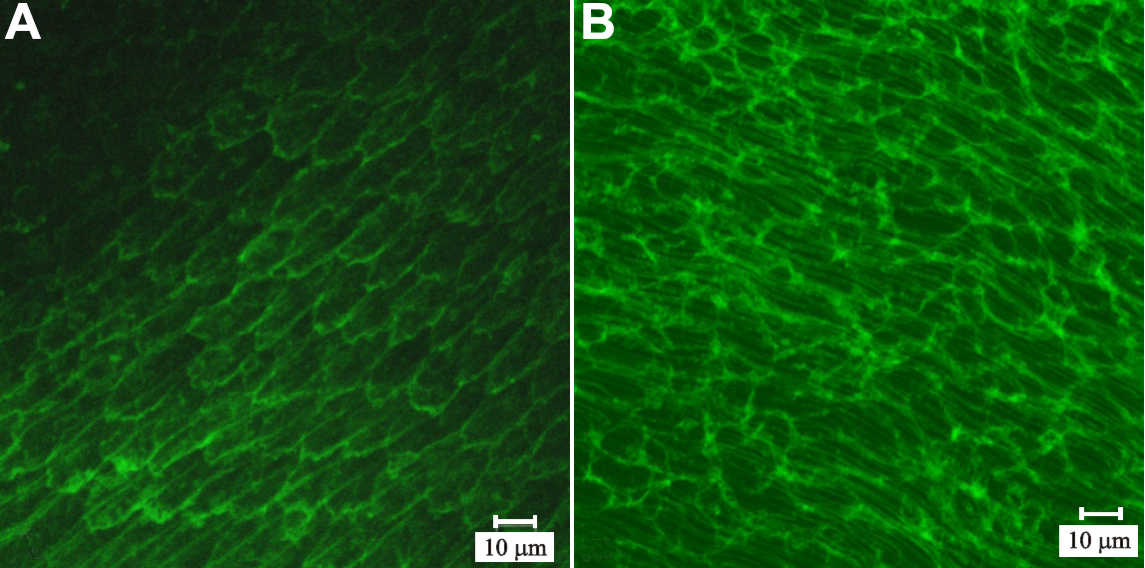
Animations of LSCM z-series from phalloidin-labeled basal fiber ends in the lateral-posterior region of fiber end migration. **Animation 1** (left): Sheared-off fiber ends adhered to the lens capsule after prefixation and decapsulation were imaged from the capsule (z=0 µm), through the CFI (z≈1 µm) and to a depth of z=5 µm. Faint F-actin fluorescence was visible at the CFI, which was gradually visualized as distinct profiles with diffuse staining in the rest of the BMC (z=2 µm through z=4 µm). By z=5 µm, lateral membrane staining of fibers was noted, indicating that in some areas, portions of elongating fibers were stripped away from the lens during decapsulation. Z-distance between frames is equal to 1 µm. **Animation 2** (right): Intact fiber ends in thick (100 µm) vibratome sections taken from the posterior lens surface. In sections taken beginning at the posterior pole, faint, scattered actin fluorescence was detected at the CFI, consistent with the presence of filopodia at this interface. Fiber end profiles were discernable by z=1µm. Lateral borders of fibers deep to the ends were detected by z=4 µm and were distinct by z=5 µm. Z-distance between frames is equal to 1.0 µm. **A** and **B** A and B show z-projections of each animation.

A prior investigation of the BMC [1] utilized a modified decapsulation technique (capsule-stripping) as well as examination of intact embryonic lenses to assess components of the BMC. In order to establish the validity of examining the BMC in Vibratome-sectioned material (i.e. absence of preparative artifacts and structural disruption), a direct comparison was made between fiber ends from decapsulated lenses and fiber ends in posterior lens sections in the present study. This comparison also provided important points of reference for interpretation of BMC component-labeling in Vibratome-sectioned material. Specifically, F-actin labeling was used to delineate fiber ends (in the posterior lateral region), then visualized in through-focus z-series’. Each z-series began at the capsule (z=0 µm) and proceeded inward through the capsule-fiber interface (CFI), the fiber feet and (if present) the lateral borders of underlying fibers. Results from the decapsulation method (Figure 3A and Animation 1) demonstrated that faint F-actin fluorescence began at the CFI (z=1µm), gradually increasing to reveal distinct fiber end profiles with diffuse staining in the remainder of the BMC. The lateral borders of an underlying elongating fiber layer was observed by z=5µm; presumably this cell layer adhered to the superficial fiber ends during the prefixation step. In posterior sections (Figure 3B and Animation 2), actin fluorescence was first detected at the CFI, with fiber end profiles rapidly becoming visible (by z=1-2µm). Eventually, the lateral borders of underlying fibers were visualized at z=5µm and were quite apparent in subsequent optical sections. In both techniques, fiber feet had a depth of approximately 4µm. Fiber end profiles in sectioned material were slightly larger and more variable in size than those seen in the decapsulation specimens, consistent with their more distal location in the lateral-posterior region of fiber end migration [27].

**Figure 4:**
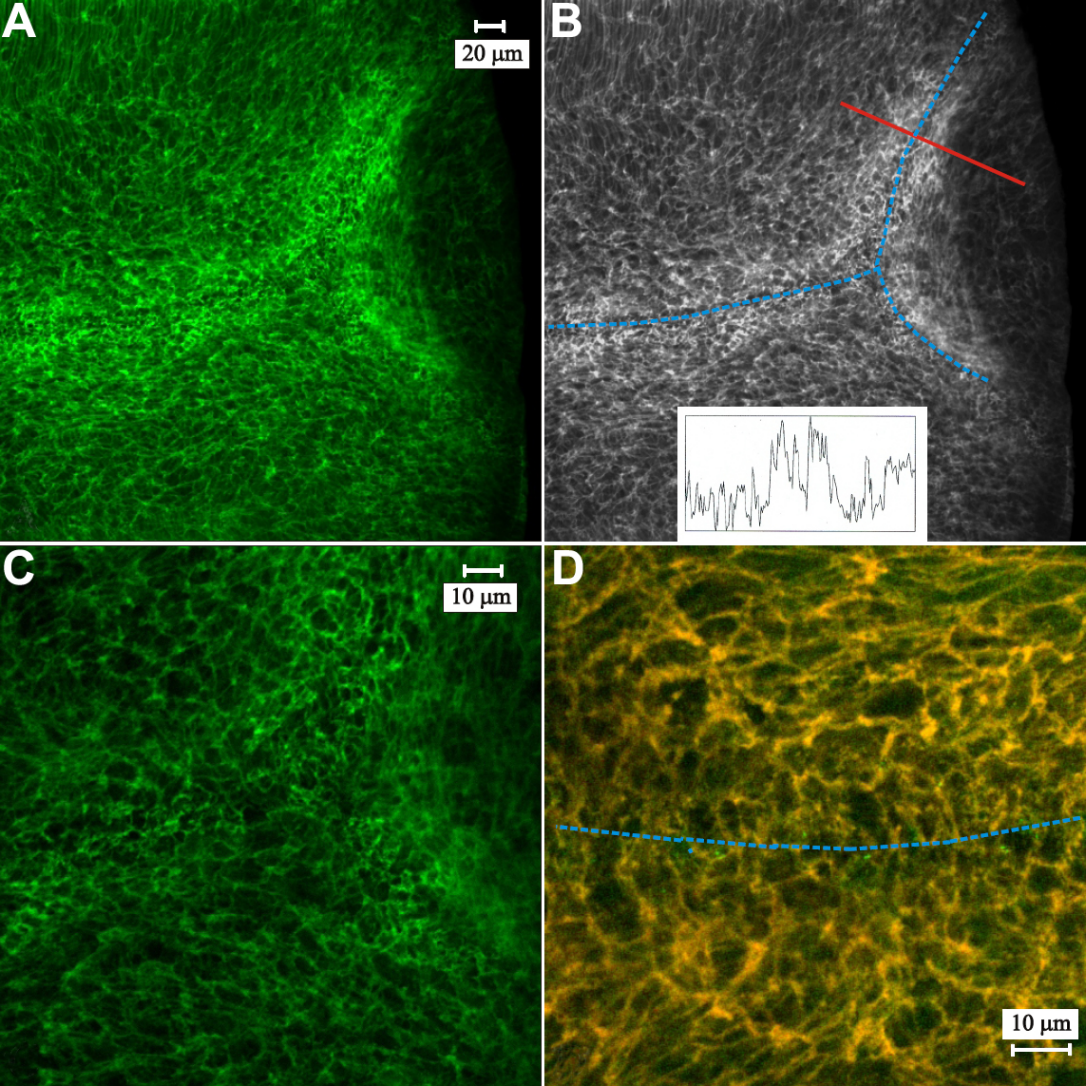
LSCM images of F-actin and fodrin distribution at, and approaching the posterior sutures. **A**: Low magnification overview of the peri-sutural and sutural regions of fiber end migration. F-actin labeling in the BMC was enhanced as migrating fiber ends approached a suture branch (left side) and forming sub-branches (right side). **B**: A greyscale version of the micrograph in **A** (identical magnification) is overlain to show the location of the posterior suture branches (blue dotted lines), the location where an optical density line scan (red solid line) was made and the resulting plot (inset). Higher labeling intensity was present within the sutural domain as demonstrated by peaks in the line scan. **C**: Higher magnification of the convergence of suture sub-branches shown in A. All BMC profiles showed strong peripheral labeling with faint fluorescence present within the brighter profiles. Fiber ends in sutural regions were rearranged into numerous smaller profiles. **D**: A merged image of double-labeling for F-actin (red) and fodrin (green) at a suture branch (blue dotted line) illustrates the extensive colocalization (yellow) of these two cytoskeletal components. The data indicates that actin is probably anchored to the membrane skeleton in the BMC at this location, which includes the sutural region and a portion of the adjacent peri-sutural region of fiber end migration.

Localization of BMC components in the peri-sutural and sutural regions was examined in 100 µm vibratome sections of normal rat lenses. Using the points of reference established in the above z-series analysis, labeling within 2 µm of the CFI was considered to be due to the BMC. As previously reported [27], F-actin labeling was predominantly localized to the periphery of the BMC with faint actin fluorescence present within the brighter profiles (Figure 4A-C). As the basal ends of the lens fibers approached the posterior suture branches, the F-actin in the BMC was rearranged into numerous smaller profiles (Figure 4C). Additionally, labeling intensity appeared to be increased at sutures. Optical density scans across sutural regions consistently showed an increased level of fluorescence (Figure 4B; inset). Because prior evidence indicated that actin was extensively associated with fodrin in fiber end segments [30], the distribution of fodrin was examined in the BMC. Double labeling for F-actin and fodrin demonstrated a very high degree of colocalization in the BMC; both in the sutural and peri-sutural regions (Figure 4D).

**Figure 5:**
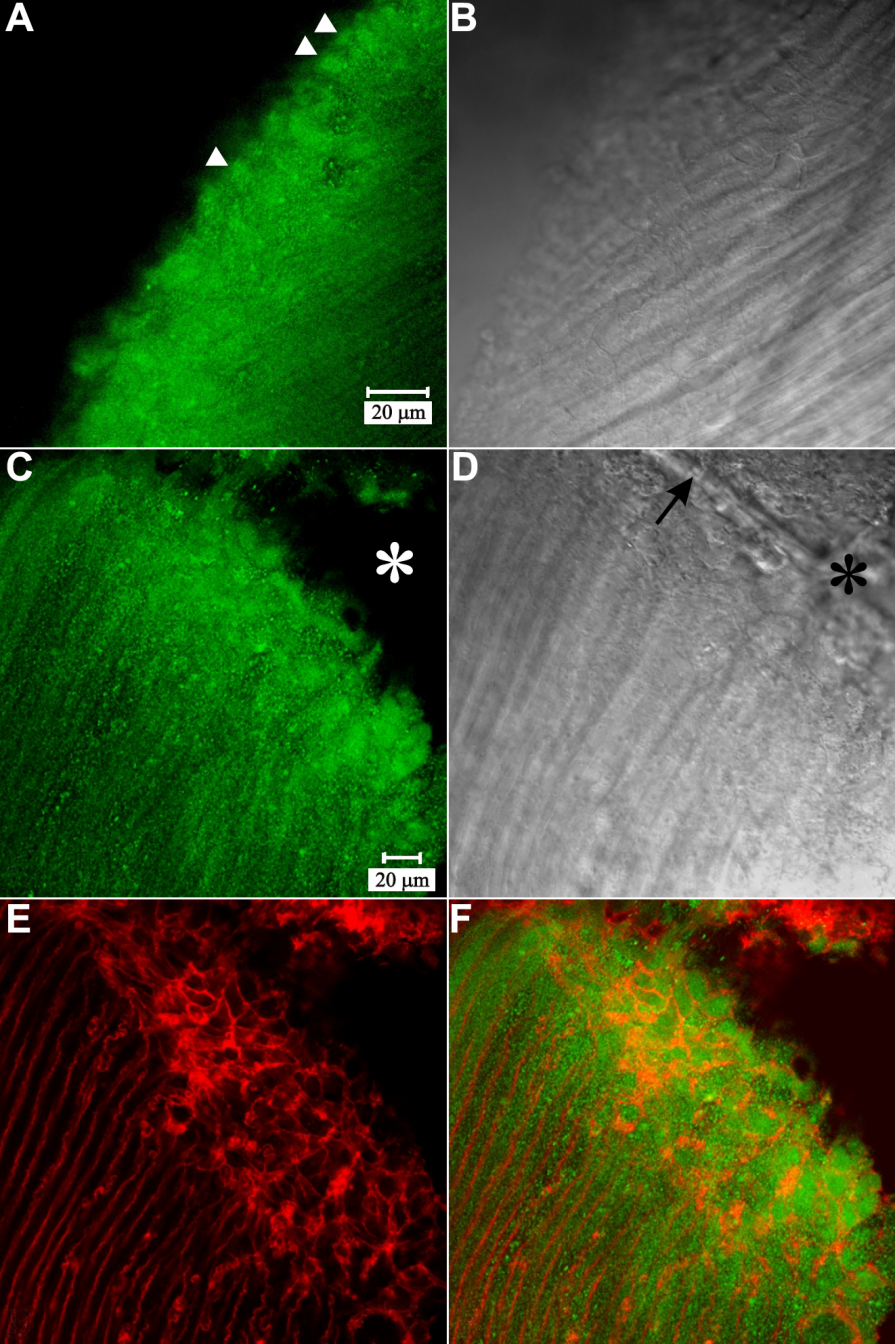
LSCM micrographs of myosin IIA and actin immuno-fluorescence at posterior fiber ends. **A**-**B**: Paired fluorescent (**A**) and DIC (**B**) images of myosin IIA labeling in the peri-sutural region. Myosin was localized as a diffuse plaque in the BMC (arrowheads) and was also present filling the cytoplasm of posterior fiber segments. **A** and **B** are at identical magnification. **C**-**F**: The same field of view showing myosin IIA fluorescence (**C**), the DIC image (**D**), actin fluorescence (**E**) and the merged myosin-actin fluorescence (**F**) at a suture branch. Basal fiber ends at the suture (**D**, arrow), were delineated by actin (**E** and **F**, red profiles) and were filled with myosin (**C** and **F**, green plaques). Asterisks (**C** and **D**) indicate an area devoid of both labels, which was due to an artifactual break in the vibratome section. **C** through **F** are at identical magnification. The data revealed that myosin IIA distribution in the BMC was consistent in the sutural, peri-sutural, and lateral-posterior regions of fiber end migration.

Labeling for myosin IIA showed abundant cytoplasmic fluorescence in posterior portions of fiber cells (Figure 5A,C,F). In the peri-sutural region, myosin was present in the BMC as a diffuse plaque, which filled the fiber ends (Figure 5A; arrowheads). Myosin distribution in the BMC was not altered in sutural regions (as compared to peri-sutural regions). Specifically, double labeling for myosin and actin (Figure 5C-F) showed fiber profiles with strong peripheral actin labeling, which were filled with diffuse myosin label.

**Figure 6:**
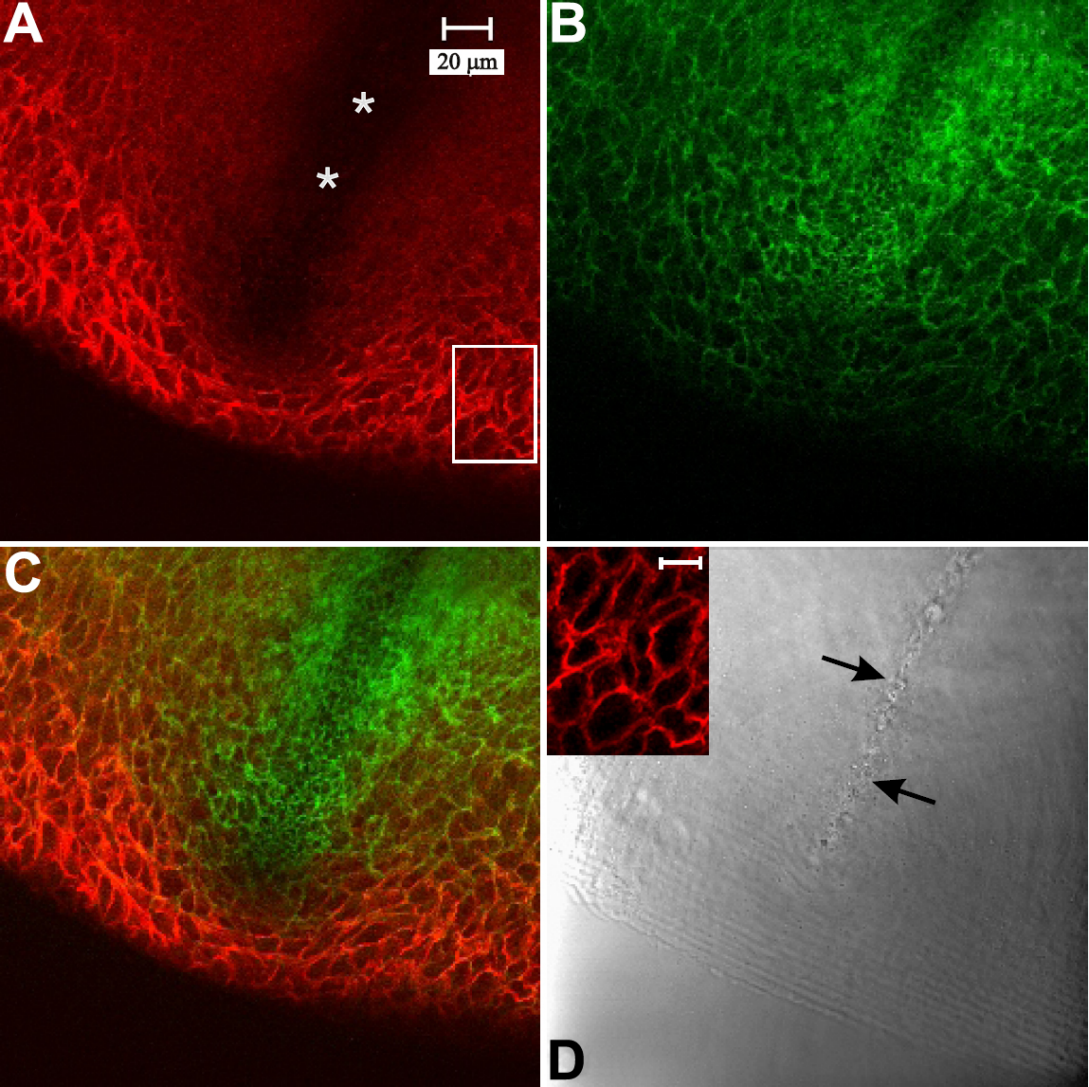
LSCM images of double-labeling for N-cadherin and F-actin at a posterior suture branch. Both N-cadherin (**A**, inset in **D**) and F-actin (**B**) were present at the margins of the BMC in the peri-sutural region. The merged image (**C**) of N-cadherin (red) and F-actin (green) fluorescence showed that they were largely co-localized at cell borders (yellow to orange coloration). However, in the sutural region, immunofluorescence for N-cadherin was conspicuously decreased (asterisk in **A** and sutural region in **C**). The location of the suture branch is indicated in the DIC image (**D**, arrows). **A** through **D** are at identical magnification. Inset is a higher magnification of boxed area in panel **A** (bar=10 µm) showing N-cadherin labeling in the BMC and demonstrates that N-cadherin was distributed fairly evenly around the BMC periphery.

**Figure 7:**
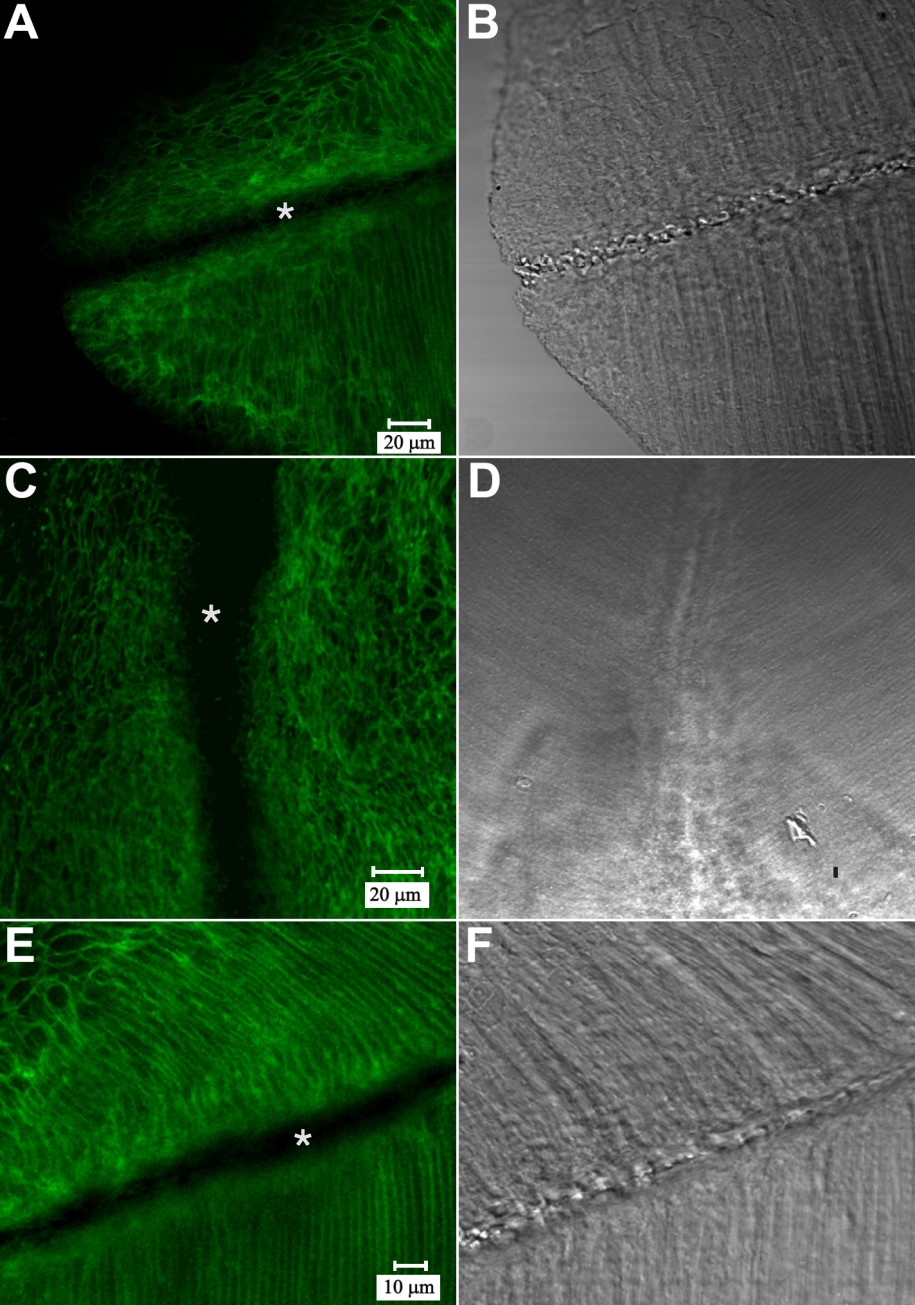
LSCM visualization of pan-cadherin immuno-fluorescence at a posterior suture branch. Paired fluorescent (**A**) and DIC (**B**) images of an oblique optical section. **A**: Because the lens surface is curved, this optical section demonstrates fluorescence due to cadherin family proteins in both the BMC (upper portion) and in the lateral fiber membranes (lower right). Notably, fluorescence is absent from the sutural region (asterisk) **A** and **B** are at identical magnification. **C**-**D**: Paired fluorescent (**C**) and DIC (**D**) LSCM images of a posterior suture branch in a detergent-extracted section. Unmasking of antigenic sites via this technique failed to reveal cadherin labeling at, and adjacent to the posterior sutures (asterisk). **C** and **D** are at identical magnification. **E**-**F**: Paired fluorescent (**E**) and DIC (**F**) images of fully-elongated fibers that have detached from the capsule and abutted to form a suture branch. Although the posterior tips of these maturing fibers lack cadherin (asterisk), strong cadherin fluorescence was apparent on the lateral fiber membranes flanking the region where fiber ends abut and interdigitate. **E** and **F** are at identical magnification.

As expected, N-cadherin immuno-fluorescence was localized at the periphery of the BMC in the peri-sutural region (Figure 6A,C). Higher magnification revealed that labeling for cadherin was distributed around the entire border of the BMC (Figure 6D; inset). Additional experiments demonstrated the same marginal pattern of N-cadherin labeling within the BMC in the lateral-posterior region (data not shown). Double labeling for N-cadherin and F-actin demonstrated that they were co-localized at the BMC border during fiber end migration (Figure 6A-D). However, at and approaching the suture branches, N-cadherin immuno-fluorescence appeared to be reduced (Figure 6A,C). Because studies have demonstrated that several cadherins are expressed in the developing lens [31,32], we utilized a pan-cadherin antibody to assess the labeling distribution of cadherin family proteins in the BMC of elongating fibers. The data showed the same distribution around the periphery of the BMC in peri-sutural regions and a clear decrease in cadherin labeling at basal fiber ends in the sutural region (Figure 7A,B), thus confirming and extending the results obtained for N-cadherin localization. Detergent extraction (to assess whether the antigenic sites were masked) demonstrated a comparable labeling pattern as in the tissue fixed and immunostained by standard techniques. That is, there was a distinct lack of cadherin labeling at, and directly adjacent to the sutural region (Figure 7C,D). In fully elongated fibers that had detached from the capsule (Figure 7E,F), cadherin labeling was lacking at the basal-to-basal fiber interface where fiber ends abut and interdigitate to form the suture. However, strong cadherin labeling was present along lateral aspects of posterior fiber segments flanking the abutted fiber ends. Through focus analysis of the sutural region revealed that fully-elongated fibers in the process of detaching from the capsule and fibers already interdigitated at sutures lacked cadherin in only the basal 3-4 µm.

**Figure 8:**
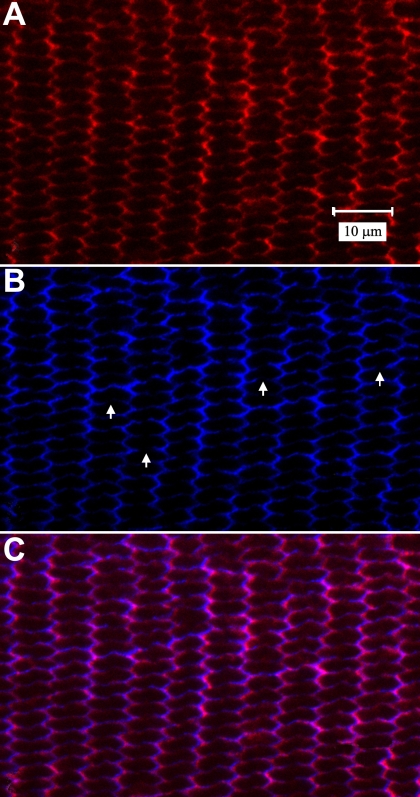
LSCM visualization of double-labeling for actin and pan-cadherin in the equatorial segments of elongating fibers. **A**: Flattened hexagonal fiber cross-sections showed actin (red) concentrated along the short faces with faint label along broad faces. **B**: Cadherin (blue) was more prominent along the short sides of fiber profiles whereas the broad sides demonstrated reduced and often discontinuous labeling (arrows). **C**: As expected, merged actin and cadherin fluorescence (purple) displayed a high degree of colocalization. Fibers shown are between 95 and 115 cells deep to the equatorial capsule, corresponding to fiber ends in the distal portion of the lateral-posterior region of fiber end migration. **A** through **C** are at the same magnification.

As stated above, both antibodies against cadherin showed that it was distributed around the entire periphery of the BMC. Because this data contrasts with studies of cadherin distribution in cross-sections of cortical lens fibers [33] as well as in the chick lens BMC [1], equatorial segments of elongating fibers were examined in the present study. Specifically, Vibratome sections through the lens equator were obtained and double-labeled for actin and cadherin (Figure 8). The flattened hexagonal profiles of fiber cross-sections displayed actin fluorescence concentrated along the short sides with faint label along broad sides (Figure 8A). Similarly, cadherin labeling was more pronounced along the short sides, while broad fiber faces demonstrated reduced and often discontinuous label (Figure 8B; arrows). Merged actin and cadherin fluorescence demonstrated far-reaching colocalization (Figure 8C).

**Figure 9:**
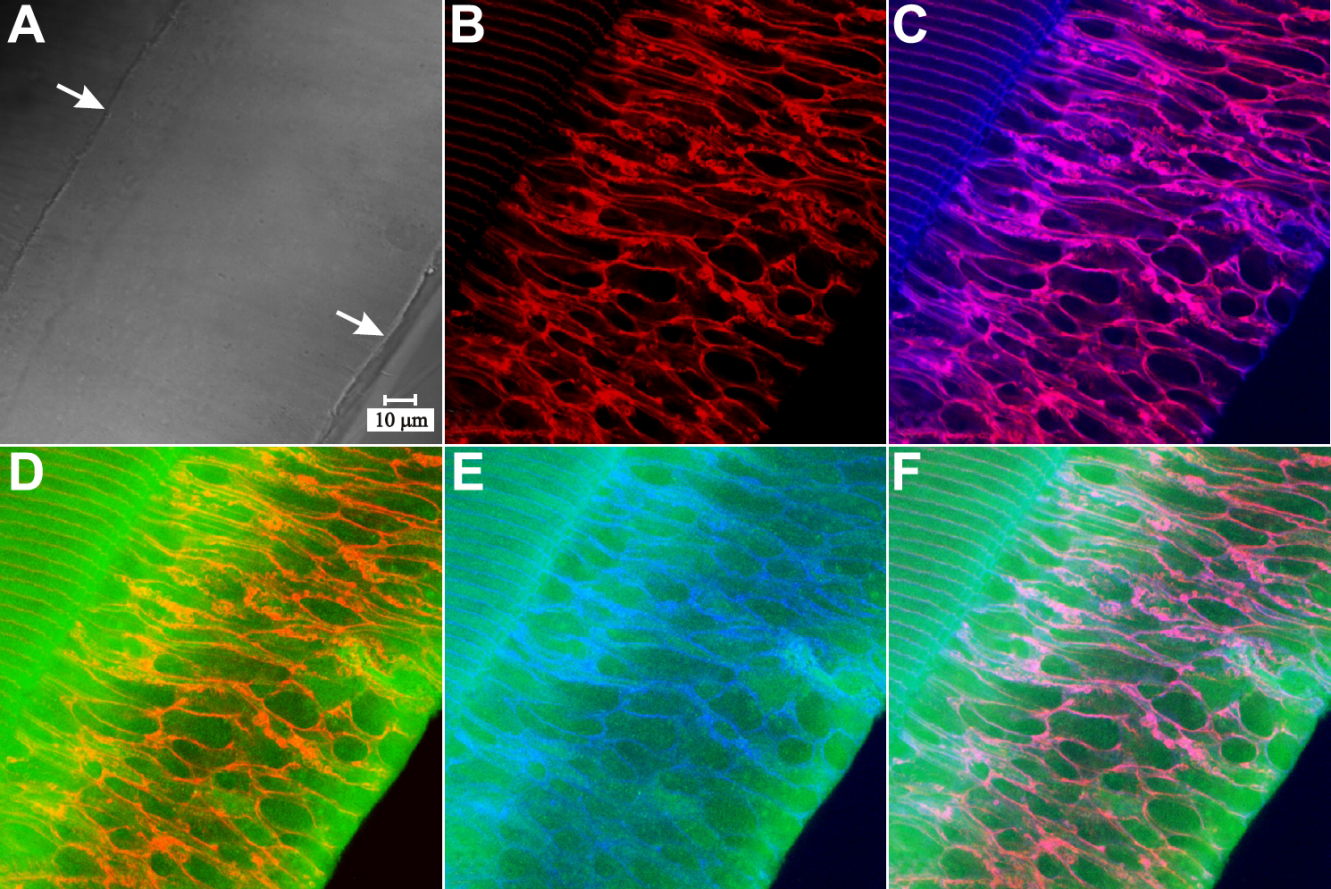
LSCM images of triple-labeling for actin, pan-cadherin and myosin IIA in the peri-sutural region. **A**: DIC image demonstrating the location of the beveled edge of the Vibratome section; the lower right arrow indicates the edge of the section and the upper left arrow indicates the extent of the bevel. **B**: Actin immuno-fluorescence (red) delineates the fiber end profiles, which are typically heterogeneous in size and shape in this region. **C**: Merged actin and pan-cadherin (blue) fluorescence demonstrates nearly complete colocalization in the BMC (purple). **D**: Merged actin and myosin IIA fluorescence (green) shows the diffuse distribution of myosin. The areas where these components are colocalized appear orange, indicating that actin predominates at the BMC periphery. **E**: Merged myosin IIA and pan-cadherin shows a comparable pattern to that in **D**, i.e. although both proteins are colocalized at the borders of profiles, the deep turquoise color indicates that pan-cadherin fluorescence is more pronounced. **F**: Merged actin, pan-cadherin, and myosin IIA fluorescence demonstrates the expected distribution. The colocaliztion of all three components appears white to pink. **A** through **F** show the same field of view and are at identical magnification.

To assess how several of the BMC components were distributed with respect to one another, triple labeling with antibodies against actin, pan-cadherin and myosin IIA was performed (Figure 9). The results were consistent with the single and double labeling results presented above. Specifically, in the peri-sutural region, both actin and cadherin fluorescence delineated fiber end profiles, while myosin was distributed diffusely throughout the fiber ends (as well as in the cytoplasm of posterior fiber segments).

**Figure 10:**
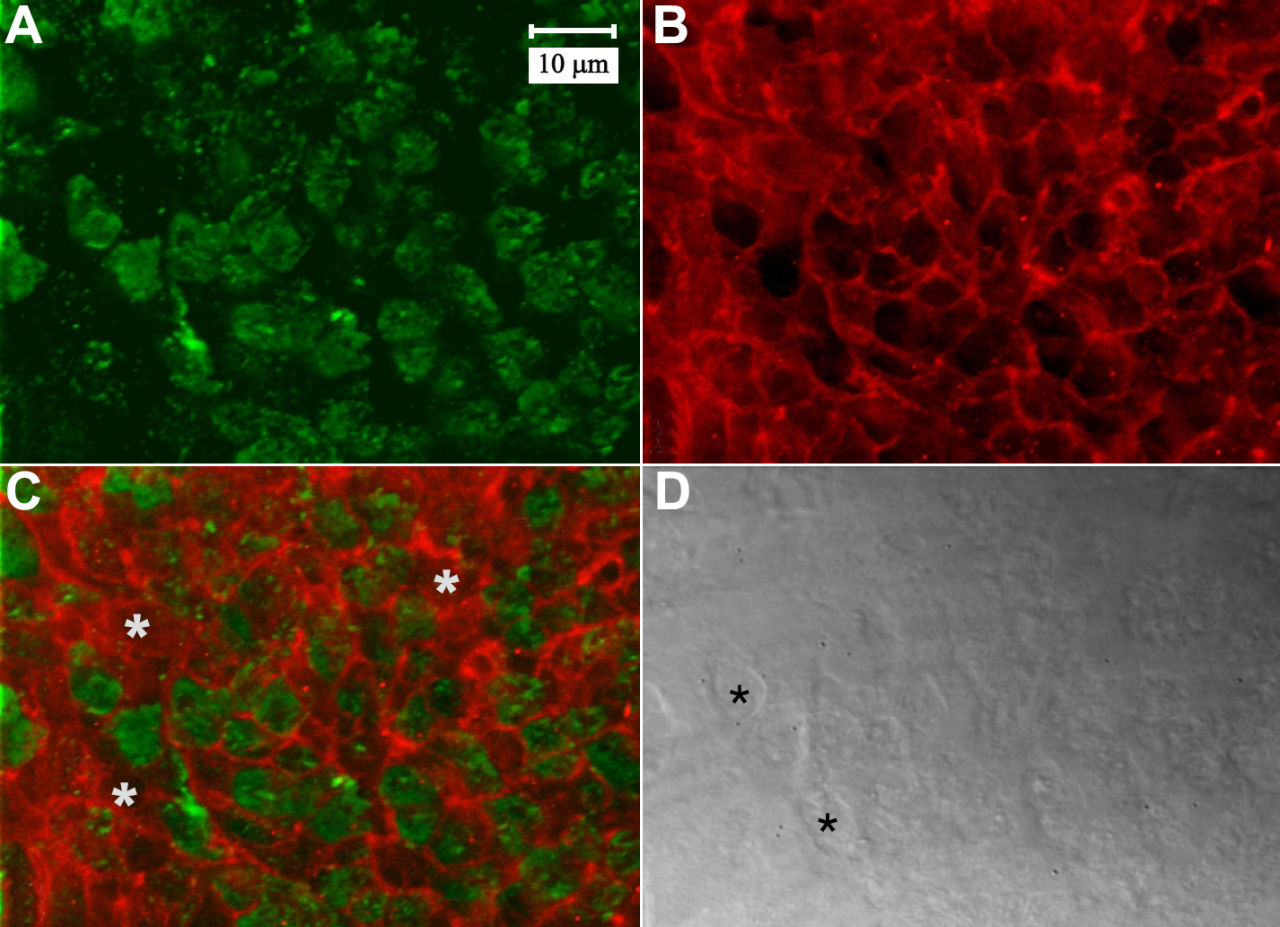
LSCM images of double-labeling for β1 integrin and F-actin in the peri-sutural region. Whereas β1 integrin (**A**) was distributed throughout the BMC as a plaque, F-actin (**B**) was most prominent at the margins of fiber ends. A merged fluorescent image (**C**) of β1 integrin (green) and F-actin (red) showed that they were not markedly co-localized. White asterisks denote profiles without integrin label. **D** shows the paired DIC image of the same field of view. Fiber ends are discernable (black asterisks). **A** through **D** are at the same magnification.

**Figure 11:**
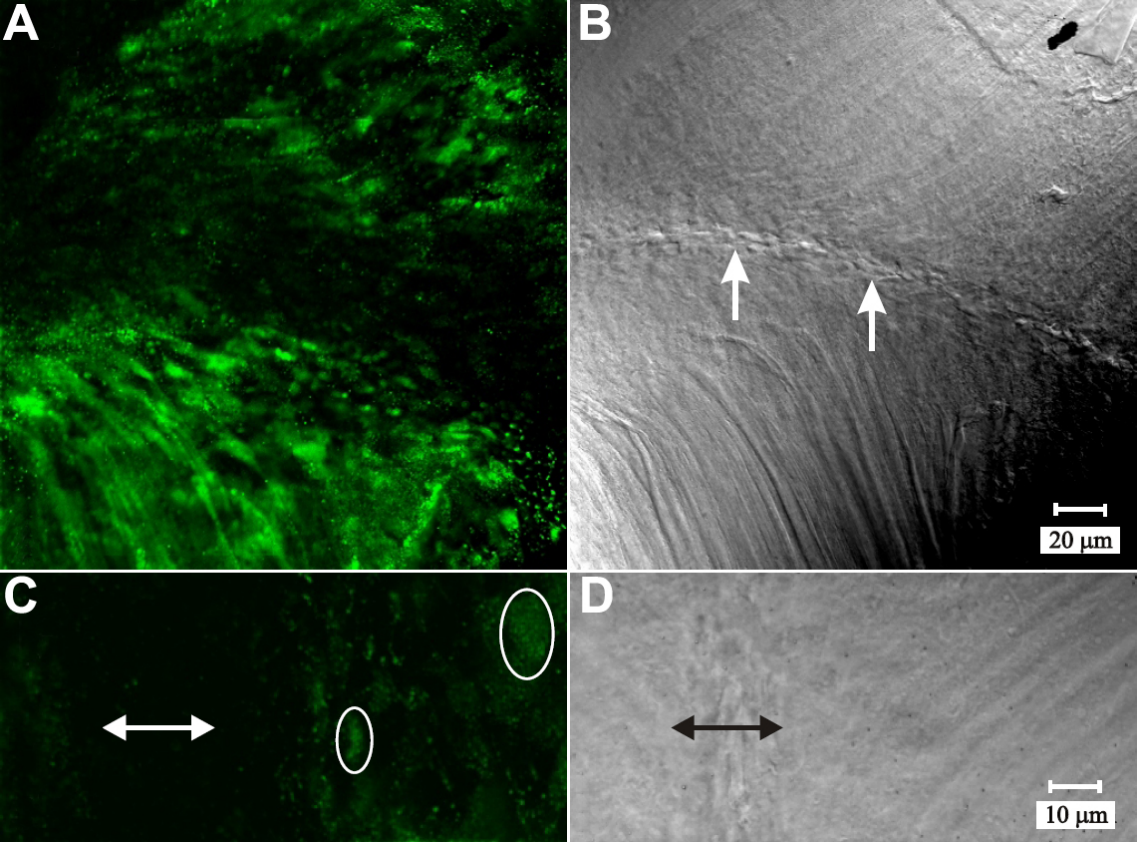
LSCM images of β1 integrin immuno-fluorescence at posterior suture branches. **A**-**B**: Paired fluorescent (**A**) and DIC (**B**) images of a posterior suture branch (arrows). The marked reduction in β1 integrin immuno-fluorescence was apparent in fiber ends at, and approaching the sutural region of fiber end migration. **A** and **B** are at identical maginificaiton. **C**-**D**: Higher magnification of paired fluorescent (**C**) and DIC (**D**) micrographs at a posterior suture branch. **C**: Approaching the suture, β-1 integrin was dispersed throughout the BMC (circled fiber end profiles). **D**: Within the nascent sutures (double-headed arrow), the paucity of label for β-1 integrin was apparent. **C** and **D** are at identical magnification.

Immuno-labeling of β-1 integrin was carried out both separately and with F-actin localization. In the peri-sutural region, localization of β-1 integrin at the CFI showed that it was distributed throughout the BMC as a diffuse plaque within the actin-rich borders (Figure 10). Absent profiles of β-1 integrin within the fiber ends were also evident (Figure 10C; white asterisks). This may be attributed to preparative procedures that may have inadvertently disrupted the adhesive properties of some of the cells. Integrin labeling was much less prominent at the posterior sutures as well as approaching the posterior sutures (Figure 11A,B). Higher magnification of sutural regions revealed that β-1 integrin labeling appeared to be markedly reduced as the lens fiber cells reach the posterior sutures (Figure 11C,D). The detergent extraction procedure had an unfortunate dampening effect on β-1 integrin labeling overall. However, sutural regions showed the same reduction in β-1 integrin labeling as specimens subjected to standard techniques (data not shown).

As negative controls, sections were treated with normal (non-immune) serum from the same species in which the primary antibody was raised in place of primary antiserum. In each case, specimens did not exhibit specific labeling (data not shown), although a low level of background was noted in some specimens.

## Discussion

The distribution of BMC components in normal rat lenses at the initiation of fiber elongation/migration demonstrated both similarities and differences with respect to that established in avian lenses in the same general region [1]. Specifically, in both models, F-actin was abundant at lateral borders of the BMC with faint actin fluorescence within the profiles. However, sheared-off fiber ends from rat lenses did not display dendritic processes around their periphery as seen in chicks. This suggests that the attachment of nascent rat lens fibers to the capsule may be less rigorous than that seen in avian lenses, since the dendrite-like extensions are probably due to partial retraction of the cell borders during the capsule-stripping technique. In addition, phalloidin-labeled basal fiber ends in rats lacked the prominent foci of actin bundles midway along hexagonal faces that were seen in embryonic chick lenses. Similar to F-actin distribution, N-cadherin was present only at the borders of the BMC in both models, but rat lenses showed a homogeneous labeling intensity around the periphery of the BMC. In contrast, N-cadherin was greatest at the midpoint of hexagonal faces in chick lenses. In both types of lenses, myosin distribution was plaque-like; however rat lenses also demonstrated strong peripheral BMC labeling whereas in chick lenses myosin appeared to be concentrated toward the center of each fiber end profile. Both models also demonstrated positive labeling for β1 integrin, but a comparison of molecular distribution in the BMC could not be made since the label was visualized from different perspectives in each case.

In rat lenses, direct comparison of basal fiber ends either attached to the capsule after decapsulation or within posterior sections, demonstrated that the two techniques result in adequate and comparable preservation of structure and BMC architecture. Thus, the BMC can be reliably located and visualized in sectioned material without the possibility of introducing mild artifacts during capsule stripping (which creates tension on the capsule-fiber linkage). Through-focus z-series analysis of intact basal fiber ends also revealed that fiber feet are ‘boutons’ having a depth of approximately 4µm from the CFI to the lateral borders of underlying fibers.

F-actin distribution in the BMC of rat lens fibers in more distal locations was consistent with that seen in equatorial region and the proximal portion of the posterior-lateral region (i.e. it was localized principally to the periphery of basal fiber ends with dim fluorescence within the remainder of the BMC). The predominantly peripheral localization of actin in the BMC suggests that actin exerts its greatest influence on cell migration at the borders of the fiber feet, possibly controlling the dynamics of leading and trailing edges of the basal membrane domain. This is consistent with evidence that various regulatory components known to be involved in actin dynamics are also present at the leading edge of migrating lens cells [34,35].

As fiber ends approached the sutural regions, the F-actin in the BMC was rearranged into numerous smaller profiles and labeling for F-actin was enhanced. As noted previously, the change in basal end morphology is consistent with the idea that fiber ends round-up and partially detach from the capsule as they approach the suture branch [27]. The increase in labeling intensity could indicate that F-actin is somewhat enriched in the BMC at the sutural regions. However, since the average size of basal fiber ends in the sutural region is approximately 1/3 that of fiber ends in the peri-sutural region [27], the increase in labeling intensity may, in part, be due to the increased number of F-actin profiles per unit area. The extensive colocalization of actin with fodrin in the BMC offers another explanation for the persistent, strong actin fluorescence in migrating basal fiber ends, despite the apparent paucity of adhesion complexes at and approaching the sutures. Fodrin’s presence and association with actin in the membrane skeleton of lens fibers has been recognized for more than twenty years [36-38] and more recent studies have shown that fodrin is present in both elongating and maturing fibers [10,29,39]. Thus, the results of the present investigation indicate that during the terminal stages of elongation, actin is likely to be anchored in the BMC via its interactions with fodrin and other membrane skeleton proteins.

N-cadherin, long known to be associated with lens fiber membranes [40,41] has since been localized to lateral fiber membranes as well as the apical and basal portions of elongating fibers [4,29,42]. It is therefore not surprising that in the present study, N-cadherin fluorescence was localized to the periphery of the BMC, coincident with the intersection of the lateral and basal membrane domains, in most regions of fiber end migration. Further, this data is somewhat consistent with results from avian lenses [1], and suggests a need for strong cell-cell adhesion during fiber end migration. It is likely that a rigorous network of cell-cell adhesions at the BMC periphery helps to maintain the established migration patterns by allowing fibers to retain their relative positions adjacent to fibers in the same growth shell and following those in the previous growth shell. In fact, the requirement for cadherins in proper fiber end migration is supported by a study of dexamethasone-treated lenses [43]. Specifically, organ-cultured rat lenses developed PSCs in which the ordered arrangement of elongating basal fiber ends was disrupted and a concurrent decrease in cadherin expression was noted following treatment.

Although cadherin was present in the BMC of chick and rat lenses, its molecular distribution was not identical. Specifically, whereas cadherin was distributed around the entire periphery of the BMC in rat lenses, it was concentrated at the midpoints of each hexagonal face in chick lenses [1]. This raises the question of whether cadherin distribution along the anterior-posterior fiber length differs between species. Prior investigations in bovine and chick lenses demonstrated that cortical fiber mid-segments have a differential distribution of both cadherin and actin to the short faces of flattened hexagonal cross-sections [1,33]. This is consistent with the results of the present investigation, which demonstrate a comparable labeling pattern in the mid-segments of elongating rat lens fibers (Figure 8). It appears that in chick and rat lenses the distribution of cadherin and actin differs in lateral as opposed to basal membranes. This underscores the idea that the BMC defines a distinct membrane domain.

In the present study, N-cadherin fluorescence in the BMC was markedly reduced approaching and within the sutural region of fiber end migration. This finding is somewhat similar to a previous investigation that showed decreased N-cadherin labeling on lateral fiber membranes soon after fibers detached from the capsule [29]. E-, N-, and B-cadherins have all been detected during lens development, however, E-cadherin is only present in lens epithelial cells [31,32]. Labeling of elongating fibers using a pan-cadherin antibody demonstrated a lack of cadherin immunofluorescence at nascent sutures in the current study. This labeling distribution persisted even after detergent extraction to uncover antigens that may have been masked by changes in molecular arrangements during the terminal stages in elongation and fiber end migration. These data indicate that the classical cadherins do not appear to be localized to the basal domains during fiber end detachment from the capsule and interdigitation to form sutures. However, the fact that cadherins (and presumably adhering junctions) were still present in the lateral membranes directly adjacent to the BMC suggests that cadherin may simply be rearranged within fiber posterior segments rather than lost. Such an arrangement would provide the necessary cell-cell contacts to maintain fiber organization both between and within growth shells.

This is the first investigation to directly show that sutures lack the appropriate adhesion molecules for adhering junctions. However, this is not surprising since, to date, the evidence supports the idea that no junctions cross the sutures. In fact, the absence of any discernable ‘junctional apparatus’ at sutures was first noted by Kuwabara in his classic study of lens ultrastructure [44]. Subsequent reports demonstrated that fibers are not connected across sutures by gap junctions [45] and that MIP is extremely sparse at apical tips of elongating fibers [46] as well as absent from their basal domains [1], making it a poor candidate for adhesion across sutures. Other common junctions, including tight junctions, desmosomes and hemidesmosomes are absent from lens fibers altogether [47-50]. The basis for adhesion of fiber ends at sutures remains unknown and may simply be comprised of structural interdigitations that provide only limited linkage between opposing groups of fibers.

In the peri-sutural region, β1 integrin was localized within the actin-rich borders of fiber ends and superimposed over the weaker F-actin fluorescence throughout the BMC. The data is consistent with previous findings that actin filaments are linked to integrins that connect the basal domain of the fiber cells to the overlying elastic lens capsule [3]. The plaque-like appearance of integrin labeling in the BMC probably reflects the arrangement of these receptors throughout the BMC, where they participate in focal contacts between the cell and matrix components in the capsule [51-55].

At and directly adjacent to the posterior sutures, labeling for β1 integrin was markedly decreased. These results contrast with a prior study that showed robust immunoreactivity for β1 integrin near the posterior pole in embryonic rat lenses [56]. The observed differences in integrin distribution may be due to temporal differences in protein distribution as a consequence of developmental stage. In the present study, detergent extraction repeatedly resulted in an overall decrease in β1 integrin labeling, however, sutural domains remained virtually empty of label, consistent with the labeling patterns noted in fixed, unextracted lens slices. While somewhat difficult to interpret, these results suggest that there may be a reduction in the overall number or type of integrin receptors that are associated with the capsule as fiber ends complete their migration and prepare to detach. One possible explanation for the decreased labeling is down regulation of integrin expression during the terminal phases of fiber end migration. In fact, studies have demonstrated that expression of some β1 integrins (α6β1B and α3β1) are down regulated during differentiation in avian lenses [2,50]. Although these changes take place early in differentiation of lens fibers, an analogous down regulation may take place during the terminal stages of fiber elongation in rats, and could underlie the change in labeling distribution in the present study. Specifically, down regulation of one sub-type of integrin receptor could result in a change in the predominant integrin type thereby creating a less adhesive attraction to the lens capsule in preparation for timely fiber end detachment.

**Figure 12:**
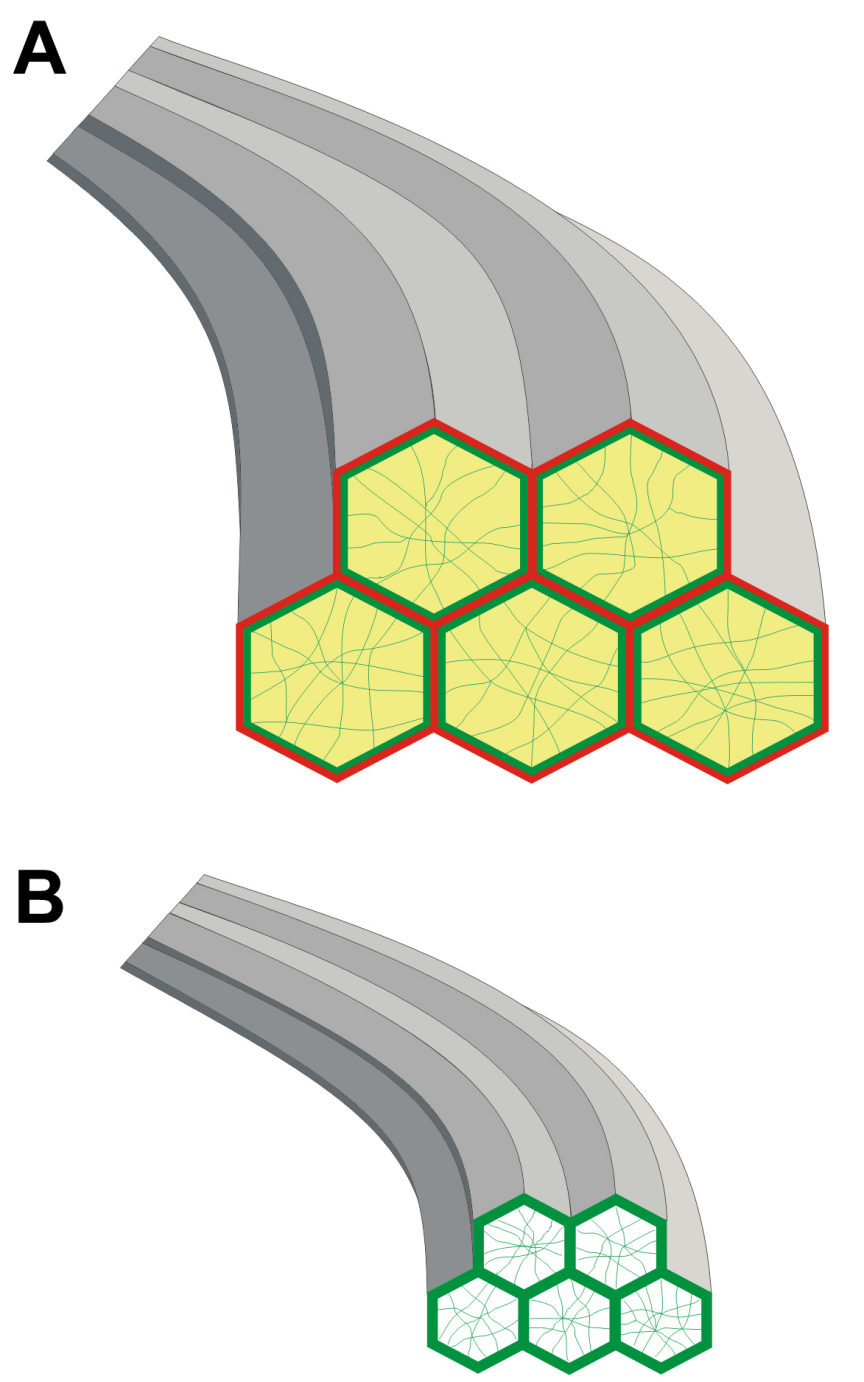
Diagram of the distribution of BMC components. **A**: In the peri-sutural region, both cadherin (red) and F-actin (green) were localized predominantly at the periphery of BMC profiles (hexagons). Some F-actin was present within the remainder of the BMC (green filaments) and β1 integrin (yellow) was localized throughout the BMC. **B**: In the sutural region of fiber end migration, peripheral staining for F-actin was somewhat enhanced, whereas neither β1 integrin nor cadherin was apparent. Myosin is not depicted because its distribution does not change as basal fiber ends detach from the capsule and interface with opposing fiber ends at posterior sutures.

The results of this investigation indicate that the distribution of some BMC components alters as fiber ends approach their sutural destinations. Specifically, F-actin, the cadherins and β1 integrin all undergo rearrangement to some degree during the final stage of fiber end migration. These changes are illustrated in a summary diagram (Figure 12). Furthermore, redistribution of BMC components appears to occur in a defined, sequential fashion, which probably facilitates the terminal migration and detachment of fibers from the capsule in order to form orderly sutures. This is significant because disruption of these processes would be likely to result in faulty migration leading to sutural malformations, which affect lens function [17,18] and in extreme cases may result in posterior subcapsular cataract formation [21,26].

The marked differences in basal fiber end morphology as well as BMC architecture between lenses having branched and branchless suture patterns have direct correlates in fully elongated fibers, in that they impact lens shape, fiber organization, and the overall sutural anatomy of each model system. Specifically, both fiber end structure and BMC architecture in chick lenses appear more organized than in rat lenses. For example, basal fiber ends in rat lenses vary significantly in size and shape as they migrate across the capsule, having the most uniform shape and basal end area just posterior to the equatorial region. However, as fiber ends progress toward their sutural destinations, uniformity is rapidly lost [27]. This contrasts with fiber ends in chick lenses, which are more regular in size and arranged in ordered rows [1,49,57]. The regular array of hexagonal fiber ends in chick facilitates the alignment of BMC components into a lattice-like arrangement that imparts contractile tone and may affect the radius of curvature of the posterior surface and even assist in accommodation [1]. Conversely, the relative disorder of basal fiber ends in the rat precludes the formation of a highly-organized molecular lattice in the BMC. This is consistent with the facts that in the rat eye, the posterior lens curvature normally remains constant [58] and active accommodation is effectively zero [59]. The superior organization of avian BMC is probably also correlated to the more rigorous adhesion of basal ends to the capsule as compared to rat. A logical corollary, since the lack of accommodation in rat lenses obviates the need to withstand the forces that would be exerted at the capsule-fiber interface during this process.

The specific migration pattern of fiber ends in a given system is a crucial factor in producing a particular sutural configuration that, in turn, affects the optical properties and focal abilities of the lens. For example, in avian lenses, all migrating fiber ends maintain their orientation directly toward the posterior pole [49,60], resulting in fibers that approximate meridians and an umbilical or branchless suture. In the rat, fibers initially migrate along meridians until they reach a latitudinal ring defined by the proximal extent of the suture branches [27]. Whereas the fiber ends of straight fibers that elongate to the proximal ends of sutures have completed their migration, the fiber ends of straight fibers that will eventually elongate to the distal end of suture branches (at the pole) continue to migrate along meridians. All other fiber ends migrate along paths that diverge from meridians after passing within the latitudinal ring such that they begin to migrate along a curved path, eventually interfacing with opposing fiber ends and aligning as longitudinal arc lengths. Thus, the variable migration paths of fiber ends within each growth shell results in opposite end curvature (S-shaped fibers) and a branched suture pattern.

In avian lenses, the contraction of the ciliary body muscles pushes the ciliary processes against the lens, compressing it into a more-rounded overall shape [61]. At the fiber cell level, the pressure of the ciliary processes forces the meridian-like fibers together at the poles, where their highly-tapered ends overlap and produce a large change in the surface curvature (lenticonus) [62]. Thus the broad accommodative range is a direct result of fiber shape and sutural anatomy (which derives from the migration patterns of elongating fiber ends). In contrast, mammalian lenses have S-shaped fibers [60]. The S-shaped fibers in primates resemble and function as simple springs, which when expanded, results in the overlap of flattened fiber ends at sutures [63]. Although the migration patterns of lenses with Y (and line) sutures result in S-shaped, simple spring fibers, they lack sufficient end taper to facilitate overlap at suture branches [62].

Although comparatively little data has, as yet, been collected on the structure and molecular components that characterize the apical fiber ends migrating across the lens epithelium, it can be assumed that at least some of the same mechanisms are involved in the organization and regulation of this process. Further study into the factors directing fiber end migration at both apical and basal fiber ends is clearly warranted.

## Supplementary Material

Animation 1

Animation 2
